# Effects of Preoperative Sarcopenia-Related Parameters on Cardiac Autonomic Function in Women with Obesity Following Bariatric Surgery: A One-Year Prospective Study

**DOI:** 10.3390/nu15122656

**Published:** 2023-06-07

**Authors:** Nara Nóbrega Crispim Carvalho, Vinícius José Baccin Martins, Vinícius Almeida da Nóbrega, Adélia da Costa Pereira de Arruda Neta, Luís Antônio Cavalcante da Fonseca, Francisco Bandeira, José Luiz de Brito Alves

**Affiliations:** 1 Department of Nutrition, Health Sciences Center, Federal University of Paraíba, João Pessoa 58010-780, Brazil; 2 Department of Endocrinology, Lauro Wanderley University Hospital, Federal University of Paraíba, João Pessoa 58050-000, Brazil; 3 Department of Physiology and Pathology, Health Sciences Center, Federal University of Paraíba, João Pessoa 58010-780, Brazil; 4 Division of Endocrinology and Diabetes, Hospital Agamenon Magalhães, Recife 52051-380, Brazil

**Keywords:** cardiac autonomic function, blood pressure, obesity, bariatric surgery, sarcopenia-related parameters

## Abstract

Objectives: Investigate changes in blood pressure (BP) and heart rate variability (HRV) in women with and without sarcopenia-related parameters who underwent bariatric surgery (BS) during a one-year follow-up. Subjects and Methods: Women were separated into obesity (OB, n = 20) and women with obesity displaying sarcopenia-related parameters (SOP, n = 14) and evaluated before BS and 3, 6, and 12 months after BS. SOP was defined as low handgrip strength (HS) and/or low appendicular skeletal mass adjusted for weight (ASM/wt × 100, %) in the lowest quartile of the sample. ASM/wt × 100, % and HS were significantly lower in SOP than OB over a one-year follow-up of BS (*p* < 0.05). Results: There was a reduction in diastolic BP, heart rate (HR), SDHR, LF, and the LF/HF ratio (*p* < 0.05) and an increase in the HF band in both groups during the follow-up period (*p* < 0.05). SOP women had reduced root mean square differences of successive RR intervals (RMSSD) and HF band and an increased LF band and SD2/SD1 ratio compared to the OB group during the one-year follow-up (*p* < 0.05). ASM/wt × 100, % was negatively associated with the LF band (r = −0.24, *p* = 0.00) and positively associated with the HF band (r = 0.22, *p* = 0.01). Conversely, HS had no association with LF (r = −0.14, *p* = 0.09) and HF (r = 0.11, *p* = 0.19). ASM/wt × 100, % and HS were negatively associated with the LF/HF ratio (*p* < 0.05). Conclusions: Women who underwent BS had an improved HRV over a one-year follow-up. However, the improvement in HRV variables was less pronounced in women with low muscle mass and/or HS during the follow-up period.

## 1. Introduction

Sarcopenic obesity is defined by high adiposity associated with low muscle mass and function, which increases the risk of disability and clinical complications [1]. The coexistence of excess adiposity, metabolic derangements, insulin resistance, and low skeletal muscle mass and function in patients with sarcopenia contributes to higher rates of cardiovascular disease, heart failure, and mortality compared to their counterparts without sarcopenia [2,3].

Young women with obesity-related diseases, such as arterial hypertension and type 2 diabetes mellitus, showed cardiac autonomic dysfunction through low heart rate variability (HRV) [4,5]. In addition, early studies have demonstrated that overweight patients with low muscle mass [6] and sarcopenic older adults [7] have lower parasympathetic tones, suggesting that the loss of muscle mass and function may be associated with poor autonomic modulation.

Enhanced sympathetic nervous activity, vagal withdrawal, and sympathovagal tone imbalances are associated with the development and maintenance of hypertension, abnormal indices of HRV, and cardiac autonomic dysfunction [8,9,10]. Autonomic nervous system (ANS) control of the heart is a dynamic process modulated by parasympathetic and sympathetic innervation. An RR variation or HRV obtained from an electrocardiogram, Holter monitor, or chest strap has been a commonly used and validated test to assess cardiac autonomic function through time, frequency, and non-linear domain indices that quantify parasympathetic (RMSSD, PRR50, HF, and SD1) and sympathetic (LF, LF/HF, and SD2) modulation [11,12,13].

Although previous studies have demonstrated low HRV in obesity and sarcopenia [4,5,6,7] and that bariatric surgery (BS) reduced blood pressure (BP) and cardiac autonomic dysfunction in patients with obesity [14,15,16,17], there are no data in the literature evaluating the impact of BS on cardiac autonomic modulation in individuals with parameters related to sarcopenia. Considering the probable loss of muscle mass after BS [18] and that irisin, a myokine that acts in neurons in the nucleus as ambiguous promoting bradycardia, is produced by skeletal muscle, it is reasonable to infer that the benefits of BS on the cardiac autonomic system might be attenuated in individuals with low muscle mass and strength. [19]. Therefore, the aim of this study was to investigate the implications of BS on the body composition, BP, and HRV variables in women with and without sarcopenia-related parameters, such as low muscle mass and/or HS, before surgery and after three, six, and twelve months of BS. The hypothesis tested was that women with positive parameters for sarcopenia before BS have a reduced improvement in cardiac autonomic function during a one-year follow-up compared to their counterparts without sarcopenia.

## 2. Subjects and Methods

### 2.1. Ethical Characteristics

The Research Ethics Committee (reference number 80984817.9.0000.5183) approved the development of the study. The study was conducted in accordance with the National Health Council and the Declaration of Helsinki. All participants gave their informed consent to be included in the study.

### 2.2. Participants and Study Design

**Figure 1:**
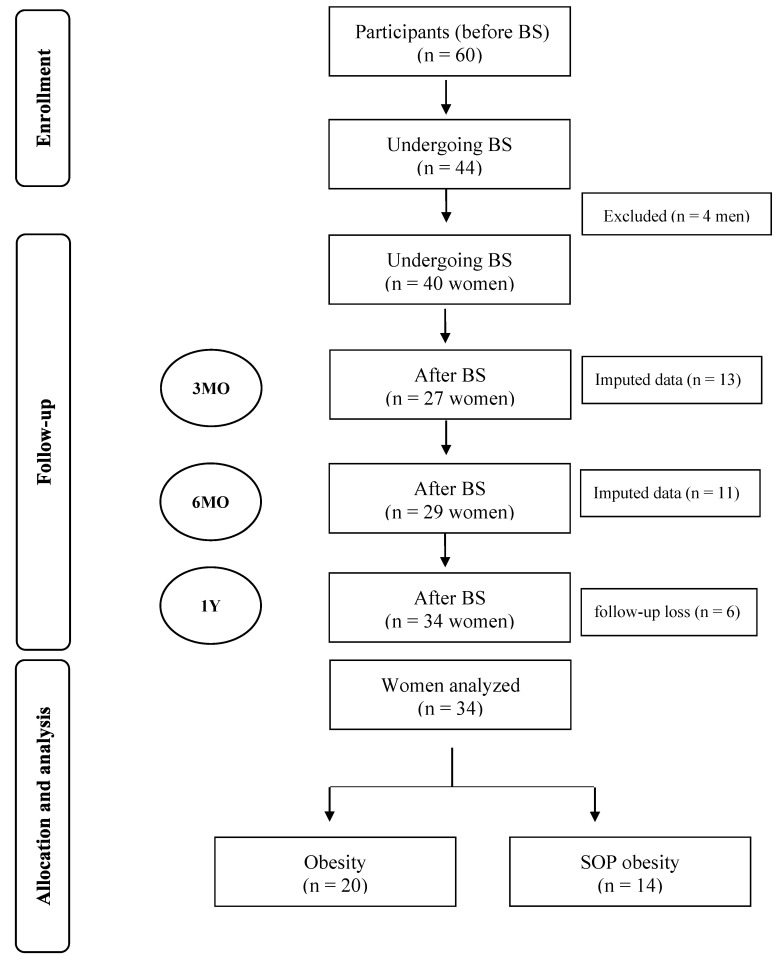
Flow chart of enrollment, follow-up, allocation, and analysis of participants before and after bariatric surgery BS: bariatric surgery; n: number; MO: months; SOP: Sarcopenic-Obesity Parameters; Y: year.

This longitudinal study was conducted on forty women. The participants were selected from the ambulatory bariatric surgery service of the Lauro Wanderley University Hospital, a hospital accredited by the public health system to perform BS (Figure 1). Sleeve gastrectomy and gastric bypass were performed by the hospital’s surgical team. Women were separated into two groups: those with obesity (OB group) and women with obesity and sarcopenia-related parameters (SOP group). The positive sarcopenia-related parameters were defined as low handgrip strength (HG) and/or weight-adjusted appendicular skeletal mass (ASM/weight × 100, %) in the lowest quartile of the sample.

Individuals of both sexes aged 18 to 60 years and with a BMI ≥ 40 kg/m^2^ or ≥35 kg/m^2^ were used as inclusion criteria. Men were excluded from our analysis because our previously published data only included women [20], only four men underwent BS, and because the neural control of the heart may differ as a function of sex [21]. The exclusion criteria were history of pregnancy, heart replacement, arrhythmias, cardiac pacemakers, and clinical reports of cardiomyopathy. Missing values at three and six months were inputted using the mean of the variable.

### 2.3. Anthropometric Measurements and Body Composition

Body weight (kg) and height (m) were measured using standardized procedures. Body composition was assessed by bioimpedance (Inbody 370, model JMW140, Chungcheong-nam-do, Korea) according to an early study [20]. The skeletal muscle mass (SMM, kg) and the fat mass (kg) of arms, legs, trunk, and body fat percentage (BFP, %) were obtained from the manufacturer’s algorithm using weight, height, sex, and age. The appendicular skeletal muscle mass (ASM/wt × 100, %) was calculated by summing the SMM of both arms and legs.

### 2.4. Muscle Function Evaluation

The dominant HS was assessed using the Jamar hand dynamometer (Sammons Preston Inc., Bolingbrook, IL, USA). An average of three trials in each hand was taken, beginning with the dominant hand, and with a pause between 30 s measurements [22].

### 2.5. Blood Pressure and Electrocardiogram Recording

The participants abstained from strenuous physical activity for 24 h prior to testing. The participants abstained from caffeinated or stimulant beverages for 36 h and from alcohol for 72 h before BP and electrocardiogram (ECG) recording [23]. ECGs were obtained from patients who had fasted for 12 h overnight. Recordings were made in the morning period. After 3 min of rest, the BP was measured with a Welch Allyn sphygmomanometer. The participants were then instructed to stand in the supine position and to breathe normally [24]. An ECG model 26T-LTS with a 5-electrode configuration was used for data acquisition (ADinstruments^®^, Bella Vista, NSW, Australia). The ECG was recorded for 10 min, and the data were exported and analyzed blindly [12,25].

### 2.6. Heart Rate Variability Analysis

Heart rate (HR) and HRV parameters were analyzed using LabChart 8 software. Time domain data such as SDRR, RMSSD, and pRR50 were examined. In addition, frequency-domain analysis (HF, LF, LF/HF ratio) of each spectral component was calculated in normalized units (un). Finally, Poincaré scatter plots were constructed and studied as a nonlinear tool to analyze nonlinear parameters (SD1 and SD2).

### 2.7. Statistical Analysis

The Shapiro–Wilk test was used to analyze normality. These data were analyzed by an independent *t*-test, Mann–Whitney or chi-squared. The body composition, blood pressure, and HRV variables were analyzed by one-way repeated measures ANOVA. The Pearson correlation coefficient (r) was used to examine the relationship between sarcopenia and HRV parameters. The SPSS 20.0 (IBM Corporation) was used for statistical analysis and *p*-value < 0.05 was used to determine significant differences between groups.

## 3. Results

### 3.1. Baseline

Baseline characteristics regarding age, anthropometric measurements, type of surgery, BP, and medical history of disease did not differ between groups. As expected, SOP women had lower ASM/wt × 100, % and dominant HS compared to the OB group before BS (Table 1).

### 3.2. Follow-Up over One Year

OB and SOP groups displayed reduced weight, BMI, BFP, and HS during the one-year follow-up after BS (*p* < 0.05). ASM/wt × 100, % and HS were significantly lower in SOP than in OB during the one-year follow-up of BS (*p* < 0.05) (Table 2).

Regarding BP and HRV variables, there was a decrease in diastolic BP, HR, SDHR, LF, and LF/HF (*p* < 0.05) and an increase in the HF band in both groups during 1 year of BS follow ups (*p* < 0.05, Table 3). Comparing the two groups, SOP women had a more reduced RMSSD and HF band and a more increased LF band and SD2/SD1 ratio than the OB group during the evaluated follow-up period (*p* < 0.05, Table 3).

**Figure 2:**
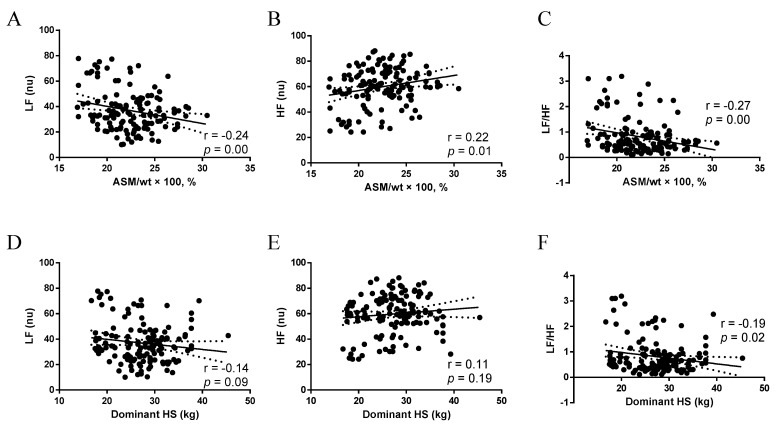
(**A**) Association between ASM/wt × 100, % and LF; (**B**) Association between ASM/wt × 100, % and HF; (**C**) Association between ASM/wt × 100, % and LF/HF; (**D**) Association between Dominant HS and LF; (**E**) Association between Dominant HS and HF; (**F**) Association between Dominant HS and LF/HF. ASM/wt × 100, %: appendicular skeletal mass adjusted for weight, LF nu, normalized unit in the low-frequency band; HF nu, normalized unit in the high-frequency band; Dominant HS: Dominant handgrip strength.

ASM/wt × 100, % was negatively associated with the LF band (r = −0.24, *p* = 0.00) (Figure 2A) and positively associated with the HF band (r = 0.22, *p* = 0.01) (Figure 2B). Conversely, HS had no association with LF (r = −0.14, *p* = 0.09) (Figure 2D) and HF (r = 0.11, *p* = 0.19) (Figure 2E). ASM/wt × 100, % and HS were negatively associated with the LF/HF ratio (*p* < 0.05) (Figure 2C,F).

## 4. Discussion

This prospective longitudinal study demonstrated that women with or without sarcopenia-related parameters and low muscle mass and/or strength had weight loss and improved BP and HRV during a one-year follow-up of BS. Additionally, this is the first study to demonstrate that the presence of low muscle mass and/or strength in the preoperative period attenuates the improvement in cardiac autonomic function compared to women without sarcopenia.

The measurements of HRV in the time and frequency domain and nonlinear analysis have been used to evaluate autonomic modulation. In the time domain, RMSSD and pRR50 have been directly related to vagal or parasympathetic activity [11]. In the frequency domain, HF spectral power is a well-known marker of parasympathetic tones, while the LF power reflects both sympathetic and vagal influences [11]. Lastly, in the Poincaré plot indices, SD1 has been correlated with short-term heart rate variability and is mainly influenced by parasympathetic modulation, while SD2 is a measure of long-term variability and reflects sympathetic activation [11,26,27].

Early studies have reported cardiac autonomic dysfunction and low HRV in patients with obesity, as indicated by the downregulation of vagal tone and sympathetic overactivity [5,28,29]. On the other hand, weight loss through lifestyle changes [30] or BS has improved HRV parameters [16]. This benefit is more pronounced in individuals with a weight loss of about 10% and is considered to be the main cause of increased parasympathetic modulation and decreased sympathetic modulation [16,30,31].

In agreement with previous studies, HRV indices increased after BS, suggesting improved autonomic control [16,32,33,34]. It has been shown that RMSSD, pRR50, HF power, and SD1, which reflects efferent parasympathetic activity, were increased in female patients in the first and third first month after sleeve gastrectomy [34]. In the present study, RMSSD, pRR50, and HF power, which may reflect parasympathetic modulation, were increased during a one-year follow-up of BS. Greater vagal activity has been reported to be cardio-protective and increase longevity [21]. In addition, LF power, which reflects the sympathetic tone, and LF:HF ratio, a measure of sympathetic-vagal tone, were decreased during a one-year follow-up of BS. Taken together, the findings may indicate an improvement in the cardiac autonomic system in women who underwent BS.

Although HRV indices have improved during a one-year follow-up of BS, it is important to highlight that women with low muscle mass and/or strength in the preoperative period showed a significantly decreased RMSSD and HF power compared to their counterparts without sarcopenia-related parameters. In addition, we found that there was a significant increase in LF power and SD2/SD1 ratio in the SOP group. Taken together, these findings showed that women with low muscle mass and strength before BS had reduced parasympathetic and increased sympathetic modulation during a one-year follow-up of BS. In fact, this observation can be reinforced by correlation analysis, in which ASM and HS were negatively associated with the LF/HF ratio, suggesting that muscle mass and function are depleted in autonomic dysfunction.

The findings of the study may be useful for clinical practice and suggest that muscle mass and strength should be measured regularly in women with obesity before BS. Furthermore, the results show that maintaining muscle mass and strength in the preoperative period may promote HRV improvement in women after BS.

The underlying mechanism by which BS improved cardiac autonomic function has not been examined in the present study, but some factors such as caloric restriction, insulin resistance, blood glucose levels, leptin, GLP-1, and N-terminal pro-brain natriuretic peptide (NT-proBNP) may be involved in the improvement of HRV [16]. A reduction in insulin resistance and glycemic levels is associated with an improved HRV after BS [33]. However, some authors did not find this association [35,36].

Early studies have reported that after BS and weight loss, there is a decrease in serum leptin levels and an improvement in HRV [35,37]. In addition, the increased plasma concentration of NT-proBNP after BS has been reported to be associated with an improved HRV [38]. Here, we demonstrated that although HR was reduced in both groups, the improvements in HRV variables in the time and frequency domain and the nonlinear measures at the one-year follow-up after BS were more effective in women with preserved muscle mass and HS. The results showed that women with low muscle mass and HS in the preoperative period have a worse recovery of cardiac autonomic function (less activation of parasympathetic activity and greater activation of sympathetic activity) during a one-year follow-up after BS.

There are very few reports in the literature correlating sarcopenia with cardiac autonomic modulation. An early study conducted on elderly Brazilians showed that individuals with sarcopenia had a greater impairment of HRV in the time domain and non-linear measures than elderly people without sarcopenia. In this study, sarcopenia was defined by low muscle mass associated with low muscle strength or low physical performance [7].

The mechanisms for the association between sarcopenia and cardiac autonomic dysfunction are still unclear. However, some points may explain the association between autonomic dysfunction and sarcopenia. First, increased low-grade inflammation, a common feature in patients with sarcopenia and obesity, has been associated with a decline in muscle mass and strength and reduced HRV [3,39]. Second, increased reactive oxygen species and mitochondrial dysfunction can lead to muscle wasting, autonomic dysfunction, and heart failure [3]. Another mechanism that may play a role in patients with obesity and sarcopenia-related parameters is the irisin, which is a myokine produced by skeletal muscle that acts in neurons in the nucleus ambiguous, one of the sites responsible for controlling heart rate. An experimental study has shown that irisin can induce bradycardia, modulate vagal tone, and promote cardiovascular protection [19]. Since 2012, irisin has been studied, and its association with various cardiovascular diseases, such as arterial hypertension, atherosclerosis, and heart failure, has been investigated. Currently, irisin has been identified as a potential marker of cardiovascular disease and a therapeutic target [40].

### Potential Limitations

The number of participants is a limitation of the study. The COVID-19 pandemic stopped BS in several participants. Due to the reduced number in the sample, men were excluded from the analyses. Our sample consisted of women and extrapolation of these results to men would not be appropriate. Participants underwent different types of surgical procedures. This could be considered as a bias, but sleeve gastrectomy and Roux-en-Y gastric bypass were similar between groups.

## 5. Conclusions

Women with obesity who underwent BS had improved HRV over one year of follow ups. However, the improvement in HRV variables was less pronounced in women with low muscle mass and/or strength over the follow-up period.

## Figures and Tables

**Table 1:** Baseline characteristics in women with obesity (OB) and sarcopenia-related parameters (SOP).

Variables	OB (n = 20)	SOP (n = 14)	*p*-Value
Age	40.4 ± 8.5	39.0 ± 11.2	0.672
BMI	41 ± 4	44 ± 4	0.131
ASM/wt × 100 (%)	21.1 ± 1.8	18.6 ± 1.8	<0.0001
Dominant HS (kg)	32.5 ± 4.8	23.5 ± 3.8	<0.0001
Neck circumference (cm)	37.9 ± 3.2	39.2 ± 2.4	0.233
Calf circumference (cm)	44.9 ±4.8	42.7 ± 4.1	0.288
Weight loss (%)—3 MO ^#^	16.4 (3.3–29.5)	16.6 (11.9–38.7)	0.875
Weight loss (%)—6 MO	20.6 ± 8.0	24.2 ± 5.7	0.159
Weight loss (%)—1 Y	24.3 ± 11.5	31.0 ± 9.9	0.086
SBP (mmHg)	112 ± 13	110 ± 15	0.764
DBP (mmHg)	74 ± 9	72 ± 7	0.502
T2DM % (n)	14.3 (3)	35.7 (5)	0.285
Hypertension % (n)	52.4 (11)	57.1 (8)	0.944
Sleeve gastrectomy % (n)	23.8 (5)	28.6 (4)	0.937
Gastric bypass % (n)	76.2 (16)	71.4 (10)	0.937

Data are presented as mean ± SD, median (interquartile range), or % (n). The data were analyzed by independent *t*-test, Mann–Whitney ^#^, or chi-square. Abbreviations: BMI: body mass index; ASM/wt × 100, %: appendicular skeletal mass adjusted for weight; Dominant HS: Dominant handgrip strength; MO: months; Y: year; T2DM: type 2 diabetes mellitus; SBP: systolic blood pressure; DBP: diastolic blood pressure; T2DM: type 2 diabetes mellitus.

**Table 2:** Assessment of anthropometric, body composition and muscle function in women with obesity (OB) and sarcopenia-related parameters at baseline and following one-year post bariatric surgery.

	OB (n = 20)				SOP (n = 14)						
	Baseline	3 MO	6 MO	1 Y	Baseline	3 MO	6 MO	1 Y	*p*-Value Time	I	*p*-Value Group
Wt (Kg)	109.8 ± 13.9	91.2 ± 12.6	86.9 ± 12.4	82.7 ± 13.8	106.5 ± 11.1	86.8 ± 11.2	80.7 ± 10.6	73.1 ± 10.2	<0.001	0.483	0.125
BMI (Kg/m^2^)	41 ± 4	34 ± 3	32 ± 4	30 ± 4	44 ± 4	37 ± 4	33 ± 4	29 ± 4	<0.001	0.060	0.377
FFMI	19.8 ± 1.9	19.1 ± 1.7	19.0 ± 1.7	18.7 ± 1.9	20.4 ± 1.8	18.9 ± 1.7	18.3 ± 1.5	18.1 ± 1.8	<0.001	0.120	0.663
BFP (%)	49.5 ± 5.1	45.9 ± 3.7	41.1 ± 5.5	39.2 ± 7.1	53.3 ± 4.9	48.6 ± 3.4	44.1 ± 6.3	38.0 ± 9.5	<0.001	0.134	0.204
ASM/wt × 100 (%)	21.1 ± 1.8	22.4 ± 1.2	23.9 ± 1.8	24.7 ± 2.3	18.6 ± 1.8	21.2 ± 1.8	22.2 ± 2.6	24 ± 3.5	<0.001	0.006	0.025
Dominant HS (kg)	32.5 ± 4.8	30.9 ± 3.5	30.0 ± 3.0	28.2 ± 4.3	23.5 ± 3.8	22.5 ± 4.2	22.6 ± 4.4	21.4 ± 3.6	0.002	0.400	<0.001

Data are presented as mean ± SD. Baseline and post-surgery data were analyzed by One-Way repeated measures ANOVA. Abbreviations: MO: months; Y: year; Wt: weight; BMI: body mass index; FFMI: free fat mass index; BFP: body fat percentage; ASM/wt × 100, %: appendicular skeletal mass adjusted for weight; Dominant HS: Dominant handgrip strength; I: Interaction.

**Table 3:** Assessment of blood pressure and heart rate variability in women with obesity (OB) and sarcopenia-related parameters (SOP) at baseline and following one-year post bariatric surgery.

	OB (n = 20)				SOP (n = 14)						
Variables	Baseline	3 MO	6 MO	1 Y	Baseline	3 MO	6 MO	1 Y	*p*-Value (Time)	I	*p*-Value (Group)
SBP, mmHg	110 ± 11	104 ± 10	106 ± 12	104 ± 12	110 ± 15	110 ± 18	105 ± 9	106 ± 10	0.129	0.513	0.588
DBP, mmHg	72 ± 8	68 ± 8	66 ± 11	66 ± 8	72 ± 7	69 ± 7	67 ± 7	66 ± 7	0.004	0.888	0.929
HR (bpm)	71.7 ± 1.1	63.9 ± 8.7	64.4 ± 8.0	63.4 ± 7.8	77.7 ± 8.5	63.4 ± 9.1	63.1 ± 9.6	62.9 ± 9.5	0.000	0.387	0.685
SDHR, bpm	3.11 ± 0.8	2.60 ± 0.8	2.81 ± 0.8	2.61 ± 0.6	3.35 ± 1.4	2.71 ±1.3	2.83 ± 1.8	2.60 ± 0.9	0.009	0.807	0.770
SDRR, ms	38.7 ± 15.5	41.0 ± 16.7	42.1 ± 13.3	41.7 ± 15.0	35.9 ±17.9	40.2 ± 15.7	41.7 ± 23.4	36.3 ± 11.8	0.614	0.597	0.789
RMSSD, ms	32.5 ± 17.1	41.1 ± 19.8	41.9 ± 19.7	48.1 ± 29.4	25.7 ± 18.3	31.2 ± 14.9	33.1 ± 16.2	30.7 ± 17.5	0.226	0.485	0.043
PRR50, ms	17.2 ± 16.7	21.4 ± 19.6	24.5 ± 22.6	24.2 ±20.2	8.2 ± 12.8	12.7 ± 13.6	14.7 ± 15.4	13.9 ± 17.3	0.531	0.994	0.071
LF, nu	42.8 ± 18.4	32.6 ± 10.9	31.9 ± 12.1	30.4 ± 11.1	54.8 ± 16.4	40.5 ± 16.1	40.6 ± 18.8	36.9 ± 17.2	0.007	0.892	0.020
HF, nu	55.7 ± 17.1	64.3 ± 11.2	62.2 ± 14.7	64.8 ± 12.2	44.1 ± 15.4	57.2 ± 15.9	56.5 ± 17.0	60.3 ± 15.8	0.005	0.802	0.056
LF/HF	1.03 ± 0.95	0.58 ± 0.29	0.65 ± 0.50	0.64 ± 0.48	1.60 ± 0.91	0.95 ± 0.89	1.04 ± 0.99	0.76 ± 0.60	0.003	0.295	0.096
SD1, ms	22.9 ± 12.1	28.1 ± 14.6	31.1 ± 15.9	32.5 ± 19.7	18.3 ± 12.8	24.0 ± 12.2	23.2 ± 11.0	21.9 ± 12.2	0.260	0.748	0.071
SD2, ms	49.2 ± 19.0	48.9 ± 20.2	50.9 ± 15.7	48.2 ± 12.9	46.3 ± 23.1	49.5 ± 20.3	52.8 ± 31.0	46.0 ± 12.7	0.554	0.994	0.901
SD2/SD1	2.47 ± 0.90	1.92 ± 0.61	1.95 ± 1.02	1.72 ± 0.50	2.91 ± 1.04	2.30 ± 1.18	2.43 ± 1.05	2.53 ± 1.21	0.092	0.480	0.024

Data are presented as mean ± SD. Baseline and post-surgery data were analyzed by one-way repeated measures ANOVA.

## Data Availability

The data that support the findings of this study are available from the corresponding author upon reasonable request.

## Abbreviations

ASM/wt × 100, %: appendicular skeletal mass adjusted for weight; BP: blood pressure; Circ: circumference; BFP: body fat percentage; BMI: body mass index; BS: bariatric surgery; DBP: diastolic blood pressure; Dominant HS: Dominant handgrip strength; FFMI: free fat mass index; HF nu: normalized unit in the high-frequency band; HR: heart rate; HRV, heart rate variability; LF nu: normalized unit in the low-frequency band; OB: obese group; pRR50: number of pairs of successive normal-to-normal beat intervals that differed by 50 ms; RMSSD: square root of the mean squared differences of successive RR intervals; SBP: systolic blood pressure; SDHR: standard deviation of heart rate; SDRR: standard deviation of successive RR intervals; and SOP: sarcopenia-related parameters. MO: months; Y: year; SBP: systolic blood pressure; DBP: diastolic blood pressure; HR: heart rate; SDHR: standard deviation of heart rate; SDRR: standard deviation of successive RR intervals; LF/HF ratio; SD: standard deviation of instantaneous; and I: Interaction.
